# Meeting Report: The Seventh Conference of the Hellenic Society for Computational Biology and Bioinformatics

**DOI:** 10.5936/csbj.201303006

**Published:** 2013-06-16

**Authors:** Stavros J Hamodrakas, Ioannis Iliopoulos, Christos A Ouzounis, Pantelis G Bagos, Vasilis J Promponas

**Affiliations:** a Faculty of Biology, Department of Cell Biology and Biophysics, University of Athens, Athens, Greece; b Division of Medical Sciences, University of Crete Medical School, Heraklion, Greece; c Institute of Applied Biosciences, Centre for Research & Technology Hellas (CERTH), Thessaloniki, Greece; d Donnelly Centre for Cellular & Biomolecular Research, University of Toronto, Toronto, Ontario, Canada; e Department of Computer Science and Biomedical Informatics, University of Central Greece, Greece; f Bioinformatics Research Laboratory, Department of Biological Sciences, University of Cyprus, P.O. Box 20537, 1678 Nicosia, Cyprus

The Hellenic Society For Computational Biology and Bioinformatics (HSCBB) was conceived as a trans-national society, representing two countries that share the same language and history, Cyprus and Greece. The first, unofficial meeting was held in Heraklion in 2006, followed by Athens in 2007 and Thessalonica in 2008. After the formation of the Society and its ‘inaugural’, fourth scientific conference in Athens during 2009, the HSCBB organized two meetings in Alexandroupolis and Patras, with the aspiration to provide the necessary exposure of graduate students to new developments, expand its horizons towards the international community, promote the development of the field and formulate a solid agenda for the future, all consistent with its primary goals (http://www.hscbb.gr). The HSCBB12 meeting, which took place in Heraklion confirmed that, even in a period of austerity and societal crisis, the local community is increasingly active.

Thanks to the support of national and international sponsors, including the Computational and Structural Biotechnology Journal, the HSCBB12 has been an unprecedented success. Approximately 100 participants have registered, providing for the first time a much clearer picture of activities of the bioinformatics communities in the two countries and material for a mapping exercise undertaken by the HSCBB, as a vehicle for further growth. One verdict from this analysis has been that today, unlike seven years ago, all major academic centers in the two countries have acquired some bioinformatics activity, with a wide range of expertise, much depending on the local needs of the corresponding organizations.

Four keynote speakers provided a wider perspective of translational bioinformatics in their own special fields: Manolis Dermitzakis (University of Geneva) on human population genomics, Anton Enright (European Bioinformatics Institute) on miRNA computational genomics, Alice McHardy (Max-Planck-Institut für Informatik) on human influenza A virus evolution, and Alexis Stamatakis (Heidelberg Institute for Theoretical studies) on computational challenges in biodiversity informatics. Moreover, another four invited speakers covered diverse, yet more practical areas in the field: Graham Ball (Nottingham Trent University) on pathway modelling, Guy Cochrane (European Bioinformatics Institute) on the European Nucleotide Archive, Ioannis Kontodinas (Biomax Informatics) on knowledge management for systems biology, and Kitsos Louis (IMBB-FORTH) on the role of ontologies in modern biology. All these contributions were highly appreciated by their audiences.

The topics covered by the conference included the following areas:

- structural bioinformatics (interaction site analysis, sequence-based structural classification, molecular recognition feature analysis);
- sequence analysis (transcriptome analysis, SNP analysis, structural variomics);
- systems biology (stochastic modeling of protein dynamics, metabolomics, epigenomics);
- gene regulation and miRNA (miRNA profiling, miRNA duplex prediction, miRNA ontologies);
- algorithms, methods & tools (tree pruning algorithms, GPU accelerators, scientific workflow management systems);
- applied/translational bioinformatics (cell signaling simulations, text mining, evolutionary bioinformatics, data integration, biomarker discovery);
- genome and comparative biology (gene duplication detection, microbial genome annotation, pangenome analysis).

The list of the 70 or so contributions (20 oral presentations, 38 posters, 4 invited presentations, 1 three-day workshop, plus the 8 keynote and invited lectures) has exceeded expectations for another reason. For the first time, contributions at the HSCBB conference had a significant number of co-authorships with multiple, highly prestigious institutions, including the EBI, the Joint Genome Institute at Berkeley, and Imperial College London.

In total, according to one study published a few years ago in the HSCBB10 proceedings, 400 papers from 63 institutions have been published in one generation, with an exponential increase during the past decade, indicating that bioinformatics have now taken root in Greece and Cyprus, with a new generation of scientists acquiring the expertise and the ability to instigate new research and education programs. According to another bibliometric study, also published in the HSCBB10 proceedings, there is still scope for growth both in quantity and quality. Greece can potentially increase the number of bioinformatics laboratories from a dozen or so today to over 30 while Cyprus might also develop bioinformatics from a handful of teams to twice that number. Moreover, the impact of HSCBB in the literature is comparable to that of Australia and New Zealand, if the GDP per capita rank of the two pairs of countries is taken into account. In other words, Greece and Cyprus have reached a level where their computational biology communities arguably have a respectable standing on the world stage. As pointed out elsewhere, the field has now grown to a point that biological research is virtually impossible without computation, so that even smaller countries do have a role in this collective effort worldwide (http://www.ploscollections.org/article/browseIssue.action?issue=info%3Adoi%2F10.1371%2Fissue.pcol.v03.i03).

We hope that the effort of the HSCBB members will ultimately generate the interest of the international community and establish a presence beyond the borders of Greece and Cyprus.

